# Vascular Endothelial Dysfunction in the Thoracic Aorta of Rats with Ischemic Acute Kidney Injury: Contribution of Indoxyl Sulfate

**DOI:** 10.1155/2022/7547269

**Published:** 2022-02-25

**Authors:** Keisuke Nakagawa, Ryosuke Tanaka, Masahide Donouchi, Masaya Kanda, Saaya Kamada, Shuhei Kobuchi, Masashi Tawa, Yasuo Matsumura, Mamoru Ohkita

**Affiliations:** ^1^Department of Pathological and Molecular Pharmacology, Faculty of Pharmacy, Osaka Medical and Pharmaceutical University, 4-20-1 Nasahara, Takatsuki, Osaka 569-1094, Japan; ^2^Division of Pharmacology, Department of Pharmacy, School of Pharmacy, Hyogo University of Health Sciences, 1-3-6 Minatojima, Chuo-ku, Kobe, Hyogo 650-8530, Japan

## Abstract

Chronic kidney disease (CKD) and cardiovascular disease are known to be linked, and the involvement of indoxyl sulfate (IS), a type of uremic toxin, has been suggested as one of the causes. It is known that IS induces vascular dysfunction through overproduction of reactive oxygen species (ROS). On the other hand, the involvement of IS in the vascular dysfunction associated with acute kidney injury (AKI) is not fully understood. Therefore, we investigated this issue using the thoracic aorta of rats with ischemic AKI. Ischemic AKI was induced by occlusion of the left renal artery and vein for 45 min, followed by reperfusion 2 weeks after contralateral nephrectomy. One day after reperfusion, there was marked deterioration in renal function evidenced by an increase in plasma creatinine. Furthermore, blood IS levels increased markedly due to worsening renal function. Seven days and 28 days after reperfusion, blood IS levels decreased with the improvement in renal function. Of note, acetylcholine-induced vasorelaxation deteriorated over time after reperfusion, contradicting the recovery of renal function. In addition, 28 days after reperfusion, we observed a significant increase in ROS production in the vascular tissue. Next, we administered AST-120, a spherical adsorbent charcoal, after reperfusion to assess whether the vascular endothelial dysfunction associated with the ischemic AKI was due to a temporary increase in blood IS levels. AST-120 reduced the temporary increase in blood IS levels after reperfusion without influencing renal function, but did not restore the impaired vascular reactivity. Thus, in ischemic AKI, we confirmed that the vascular endothelial function of the thoracic aorta is impaired even after the recovery of kidney injury, probably with excessive ROS production. IS, which increases from ischemia to early after reperfusion, may not be a major contributor to the vascular dysfunction associated with ischemic AKI.

## 1. Introduction

Ischemia/reperfusion (I/R) is one of the leading causes of acute kidney injury (AKI), with a high morbidity and mortality in hospitalized patients [1]. In addition to there being no established treatment for AKI, a major medical problem is that after AKI develops, it progresses to chronic kidney disease (CKD) and end-stage renal disease. Although many studies have been conducted on the mechanism of transition to CKD after the onset of AKI, it is not fully understood [2, 3].

Decreased vascular endothelial function leads to the development of not only renal damage but also cardiovascular disease [4]. In this regard, vascular endothelial dysfunction is known to be caused by AKI [5, 6]. AKI is accompanied by reactive oxygen species (ROS) overproduction [7], and increased ROS production is considered to lead to the inactivation and/or reduction of endothelium-derived relaxing factors, such as nitric oxide (NO) and endothelium-derived hyperpolarizing factor, resulting in vascular endothelial dysfunction [8]. Of note, vascular endothelial dysfunction associated with AKI is caused not only in the primary lesion [5] but also in distant sites [6], which is thought to create a vicious cycle of multiple organ injury. It is also possible that vascular endothelial dysfunction after the onset of AKI plays a role in the subsequent progression to CKD.

We reported that indoxyl sulfate (IS), a uremic toxin that accumulates during the progression of renal failure, promotes the generation of excess ROS in the thoracic aorta of normal rats and inactivates NO, leading to vascular dysfunction [9]. Of note, the administration of spherical adsorbent charcoal AST-120 to CKD model rats reduced blood IS levels and improved vascular endothelial function [10]. Thus, there is strong evidence and support for the involvement of IS in endothelial dysfunction in renal injury. In AKI, the blood IS concentration increases with a decline in renal function [11]. However, it is unclear whether IS is involved in the vascular endothelial dysfunction associated with AKI. In this study, we evaluated changes in vascular endothelial dysfunction and IS levels over time in ischemic AKI. Furthermore, AST-120 was administered from the onset of AKI to evaluate the contribution of IS on vascular endothelial dysfunction.

## 2. Materials and Methods

### 2.1. Animals

Male Sprague-Dawley rats (10 weeks of age, Japan SLC, Shizuoka, Japan) were used. Rats were housed in a light-controlled room with a 12 hr light/dark cycle and allowed ad libitum access to food and water. This study involving animals was approved by the Experimental Animal Committee at the Faculty of Pharmacy, Osaka Medical and Pharmaceutical University (permission code: 5, permission date: 31 March 2018).

### 2.2. Experimental Procedure

**Figure 1:**
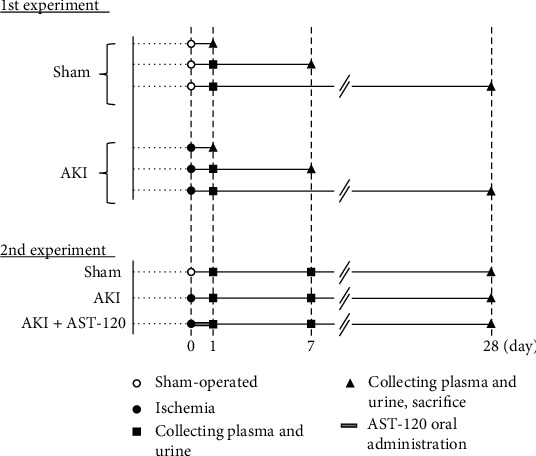
Schematic diagram depicting the experimental protocols. AKI: acute kidney injury.

Experimental protocols are shown in Figure 1. At 8 weeks of age, the right kidney was resected through a small flank incision under anesthesia. In an experiment to examine vascular endothelial function after ischemic AKI, these animals were divided into two groups: (1) sham-operated control without ischemia and (2) ischemic AKI by renal I/R. In order to induce ischemic AKI, the left kidney was exposed through a small flank incision. The left renal artery and vein were occluded with a Schwartz microserrefine. After 45 min, the microserrefine was removed, and reperfusion was visually confirmed. In sham-operated control rats, the left kidneys were treated in the same manner as those in the ischemic AKI group except for the ischemia treatment. All rats were placed in metabolic cages 1, 7, and 28 days after reperfusion, and urine was collected for 5 hr. After collection of urine, rats were injected with heparin (1000 U/kg, i.v.), and blood samples were drawn from the abdominal aorta. The collected blood and urine were used to measure renal function parameters and IS concentrations. The thoracic aorta was then removed and used for measurements of vascular function and superoxide anion (O_2_^−^) production. Rats with vascular function measurements at 7 and 28 days after reperfusion were confirmed to have developed AKI by collecting blood samples from the jugular vein 1 day after reperfusion and checking the deterioration of renal function. The same procedure was performed in the sham group.

In separate experiments, to clarify the relationship between IS and vascular endothelial function in AKI, AST-120 (Kremezin, Kureha Corporation, Japan, 2.5 g/kg, p.o.) was administered 3, 6, and 24 hr after reperfusion. The steps for creating ischemic AKI are described above and Figure 1. Blood and urine samples were collected 1, 7, and 28 days after reperfusion, and on day 28, the thoracic aorta was removed, and vascular function was measured. The collected blood and urine were used to measure renal function parameters and IS concentrations.

### 2.3. Analytical Procedure

The plasma used for measurement was collected by centrifugation of the collected blood. Plasma and urinary creatinine were measured by the Jaffe method, and creatinine clearance was calculated.

IS concentrations in plasma and urine were measured by high-performance liquid chromatography (HPLC)-fluorescence, as previously reported [12]. Briefly, a 125 *μ*L aliquot of diluted plasma and urine was added directly to 450 *μ*L of acetonitrile. After centrifugation at 10,000 rpm for 15 min, the supernatant was assayed by HPLC. The HPLC system consisted of a pump L-2130 (Hitachi, Tokyo, Japan) and FL detector L-2485 (Hitachi, Tokyo, Japan). A COSMOSIL PBr Packed Column 4.6 mml.D.^∗^250 mm (Nacalai Tesque Inc., Ltd., Kyoto, Japan) was used for the stationary phase. The mobile phase consisted of acetate buffer (0.02 M, pH 4.5)/acetonitrile. The flow rate was 1 mL/min. IS was detected using a fluorescence monitor. The excitation/emission wavelengths were 280/375 nm.

### 2.4. Vascular Function Study

Macrovascular dysfunction is well known to be coupled with cardiovascular diseases [13, 14]; therefore, we focused on the aorta in this study. The thoracic aortas of sham and ischemic AKI rats were isolated, and the connective and adipose tissues were carefully removed to make approximately 2 mm aortic ring segments. Each ring was suspended in an organ bath (10 mL) containing Krebs Ringer bicarbonate solution with the following composition (mM): NaCl 118.5, KCl 4.7, MgSO_4_ 1.2, CaCl_2_ 2.5, NaHCO_3_ 25, glucose 10, and KH_2_PO_4_ 1.2. The solution was bubbled with 5% CO_2_ and 95% O_2_ (37°C). The changes in vascular tone tension in response to vasoactive substances were continuously recorded by a polygraph system (RM 6000, Nihon Kohden, Tokyo, Japan) via an isometric force transducer (TB-612 T, Nihon Kohden). All aortic rings were precontracted with phenylephrine (Phe, 10^−6^ M). After reaching a plateau, each vasodilator (ACh [10^−9^ M -10^−5^ M)], sodium nitroprusside (SNP) [10^−9^ M -10^−5^ M]), was applied in cumulative concentrations. The vasorelaxation responses to vasodilators were presented as a percent relaxation of Phe-induced precontraction.

### 2.5. Measurement of O_2_^−^ Production in Vascular Tissues

The method for producing the aortic ring was the same as described above. Levels of O_2_·^−^ in the aortic ring were measured by measuring lucigenin-enhanced chemiluminescence using a luminometer (Lumat 3, Belthold Technologies, Schwarzwald, Germany). The aortic ring was placed in a test tube containing Krebs-HEPES buffer (99.01 mM NaCl, 4.69 mM KCl, 1.87 mM CaCl_2_, 1.2 mM MgSO_4_, 1.03 mM K_2_HPO_4_, 25 mM Na-HEPES and 11.1 mM glucose, pH 7.4, 37°C), which were bubbled with a 5% CO_2_ and 95% O_2_ gas mixture. Thereafter, lucigenin (5 *μ*M) was added to the test tube in a luminometer. The O_2_·^−^ production level was expressed in relative light units (RLU)/min/mg dry tissue weight. The luminescence in the absence of an aortic ring was used as the background and subtracted from the measured value.

### 2.6. Histological Examination of the Thoracic Aorta

The thoracic aorta was immersed in phosphate-buffered 10% formalin and embedded in paraffin. The sample was cut at 3 *μ*m and stained with hematoxylin-eosin.

### 2.7. Drugs

ACh and SNP were purchased from Sigma-Aldrich (St. Louis, MO, USA). Formaldehyde, lucigenin, and phenylephrine were purchased from Nacalai Tesque Inc., Ltd., (Kyoto, Japan). Heparin was purchased from the NIPRO Corporation (Osaka, Japan).

### 2.8. Statistical Analysis

All data represent the mean ± SEM. The unpaired Student's *t*-test was used for two-group comparisons, and a one-way analysis of variance (ANOVA) followed by Dunnett's tests was used for multiple comparisons (three groups). The least squares regression analysis was used to evaluate the relationships between variables. Differences were considered significant at *P* < 0.05.

## 3. Results

### 3.1. Renal Function after I/R

**Figure 2:**
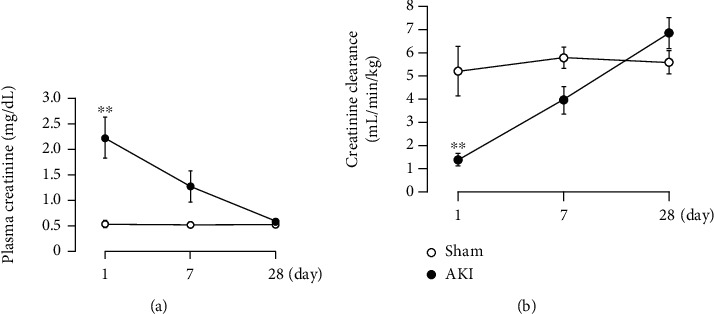
Changes in renal function ((a) plasma creatinine, (b) creatinine clearance) after renal I/R. Each point and bar represent the mean ± S.E.M. Sham-1 day, *n* = 5; sham-7 days, *n* = 5; sham-28 days, *n* = 6; AKI-1 day, *n* = 8; AKI-7 days, *n* = 10; AKI-28 days, *n* = 8. ^∗∗^*P* < 0.01, compared with sham. AKI: acute kidney injury.

Renal I/R treatment caused a significant increase in plasma creatinine (Figure 2(a)) and decrease in creatinine clearance (Figure 2(b)) at 1 day after reperfusion. Thereafter, these parameters improved over time, and at 28 days after reperfusion, renal function was similar to that of the sham-operated rats.

### 3.2. Vascular Reactivity to ACh in the Thoracic Aorta 1, 7, and 28 Days after Reperfusion

**Figure 3:**
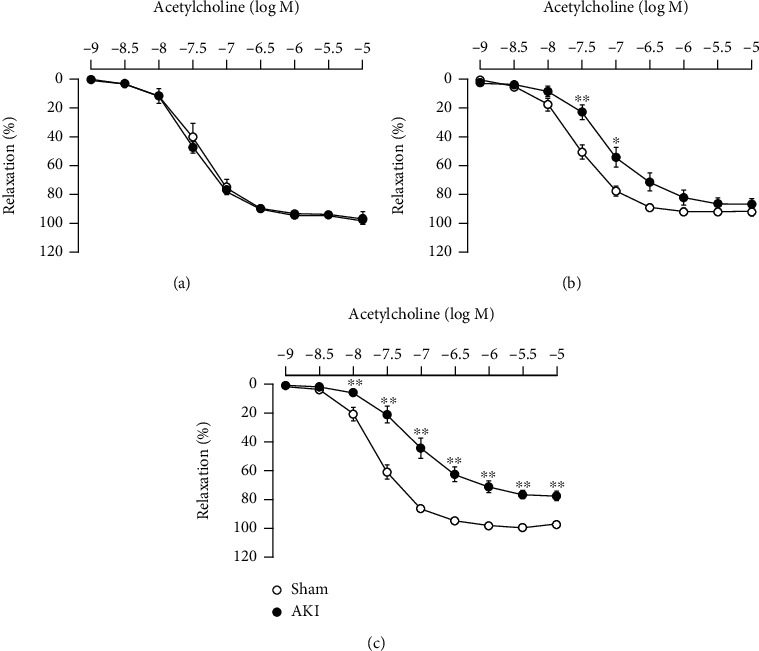
Vascular reactivity to ACh in the thoracic aorta (a) 1 day, (b) 7 days, and (c) 28 days after reperfusion. Each point and bar represent the mean ± S.E.M. Sham-1 day, *n* = 5; sham-7 days, *n* = 5; sham-28 days, *n* = 6; AKI-1 day, *n* = 8; AKI-7 days, *n* = 10; AKI-28 days, *n* = 8. ^∗^*P* < 0.05 and ^∗∗^*P* < 0.01, compared with sham. AKI: acute kidney injury.

The results after 1 day of reperfusion are shown in Figure 3(a). There was no influence of I/R treatment on the vasorelaxant response to ACh. However, as shown in Figures 3(b) and 3(c), there was a significant decrease in vascular reactivity to ACh with I/R treatment compared with the sham group at 7 and 28 days after reperfusion. In particular, the decrease was more marked 28 days after reperfusion. Throughout the period, the sham group had slightly augmented reactivity to ACh over time after surgery, but the AKI group had attenuated reactivity.

### 3.3. Vascular Reactivity to SNP in the Thoracic Aorta 28 Days after Reperfusion

**Figure 4:**
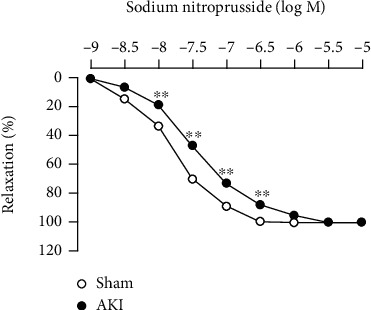
Vascular reactivity to SNP in the thoracic aorta 28 days after reperfusion. Each point and bar represent the mean ± S.E.M. Sham, *n* = 6; AKI, *n* = 8. ^∗∗^*P* < 0.01, compared with sham. AKI: acute kidney injury.

Next, we assessed endothelium-independent vascular function 28 days after reperfusion using SNP, an NO donor. As shown in Figure 4, I/R treatment reduced the vascular reactivity to SNP.

### 3.4. O_2_^−^ Production in the Thoracic Aorta 28 Days after Reperfusion

**Figure 5:**
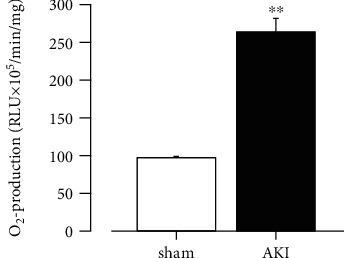
O_2_^−^ production in the thoracic aorta 28 days after reperfusion. Each column and bar represent the mean ± S.E.M. Sham, *n* = 6; AKI, *n* = 8. ^∗∗^*P* < 0.01, compared with sham. AKI: acute kidney injury; RLU: relative light units; O_2_^−^: superoxide anion.

Next, we used the thoracic aorta 28 days after reperfusion to examine O_2_^−^ production in the vascular tissue. As shown in Figure 5, there was a marked increase in O_2_^−^ production in the thoracic aorta of rats with ischemic AKI.

### 3.5. Effects of AST-120 Administration on Renal Function after I/R

**Figure 6:**
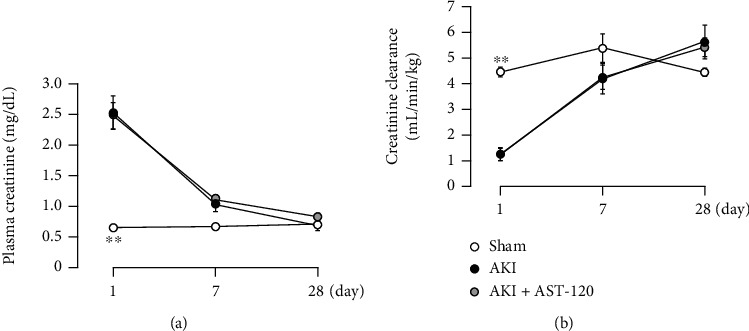
Effects of AST-120 on renal function ((a) plasma creatinine, (b) creatinine clearance) after renal I/R. Each point and bar represent the mean ± S.E.M. Sham, *n* = 5; AKI, *n* = 7; AKI + AST-120, *n* = 8. ^∗∗^*P* < 0.01, compared with AKI each day. AKI: acute kidney injury.

Similar to the results above, the renal I/R treatment caused the marked deterioration of renal function 1 day after reperfusion, followed by recovery over time. In addition, AST-120 administration did not cause significant changes in plasma creatinine (Figure 6(a)) and creatinine clearance (Figure 6(b)) at any time point after reperfusion.

### 3.6. Effects of AST-120 Administration on IS Concentration in Plasma and Urine after I/R

**Figure 7:**
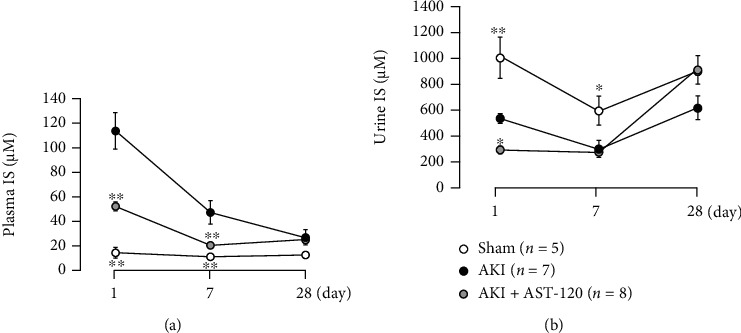
Effects of AST-120 on IS concentration in (a) plasma and (b) urine after renal I/R. Each point and bar represent the mean ± S.E.M. Sham, *n* = 5; AKI, *n* = 7; AKI + AST-120, *n* = 8. ^∗^*P* < 0.05 and ^∗∗^*P* < 0.01, compared with AKI each day. AKI: acute kidney injury; IS: indoxyl sulfate.

In the ischemic AKI group, the blood IS concentration markedly increased (Figure 7(a)), whereas the urinary IS concentration decreased (Figure 7(b)) compared with the sham group at 1 day after reperfusion; IS excretion into urine was likely suppressed by the marked deterioration of renal function. Seven and 28 days after reperfusion, the blood IS concentration decreased along with the recovery of renal function, and the urinary IS concentration slightly increased. On the other hand, AST-120 reduced IS concentrations in blood and urine at 1 and 7 days after reperfusion; although, the plasma levels 28 days after reperfusion were comparable regardless of AST-120 administration.

### 3.7. Effects of AST-120 Administration on Decreased Vascular Reactivity to ACh in the Thoracic Aorta Associated with AKI

**Figure 8:**
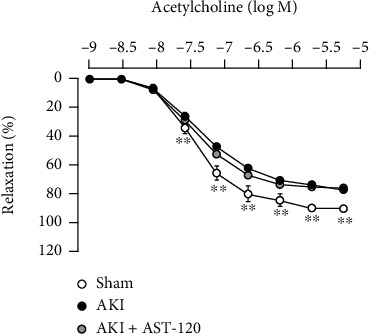
Effects of AST-120 on decreased vascular reactivity to ACh in the thoracic aorta associated with AKI. Each point and bar represent the mean ± S.E.M. Sham, *n* = 5; AKI, *n* = 7; AKI + AST-120, *n* = 8. ^∗∗^*P* < 0.01, compared with AKI. AKI: acute kidney injury.

A significant decrease in the ACh-induced vasorelaxation response was observed in the thoracic aorta 28 days after reperfusion compared with the sham group. In addition, AST-120 administration did not improve vascular endothelial dysfunction after the onset of AKI (Figure 8).

### 3.8. Effects of AST-120 Administration on Increased O_2_^−^ Production in the Thoracic Aorta Associated with AKI

**Figure 9:**
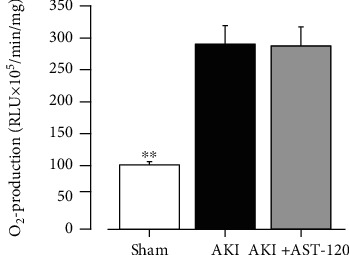
Effects of AST-120 on O_2_^−^ production in the thoracic aorta associated with AKI. Each column and bar represent the mean ± S.E.M. Sham, *n* = 5; AKI, *n* = 6; AKI + AST-120, *n* = 5. ^∗∗^*P* < 0.01, compared with AKI. AKI: acute kidney injury; RLU: relative light units; O_2_^−^: superoxide anion.

The thoracic aorta 28 days after reperfusion exhibited significant O_2_^−^ production compared with the sham group. In addition, AST-120 administration did not reduce the O_2_^−^ overproduction associated with I/R (Figure 9).

### 3.9. Association between Plasma IS Levels and Vascular Reactivity to ACh and O_2_^−^ Production in the Thoracic Aorta

We examined the association between blood IS levels 1 day after reperfusion and vascular endothelial function and O_2_^−^ production 28 days after reperfusion. Regression analyses considering the presence of I/R revealed that the plasma concentration of IS is not associated with the vasorelaxing efficacy of ACh at 10^−7^ M, a concentration close to the half maximal effective concentration. Similarly, there was no association between the IS levels and O_2_^−^ production (Table 1).

### 3.10. Histological Changes in the Thoracic Aorta 28 Days after Reperfusion

**Figure 10:**
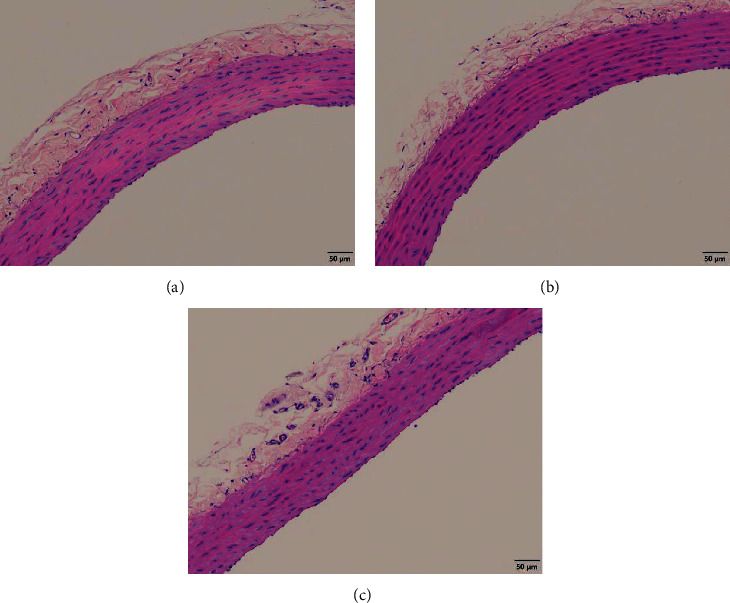
Representative images of the thoracic aorta 28 days after reperfusion stained with hematoxylin-eosin. (a) Sham, (b) AKI, and (c) AKI + AST-120. AKI: acute kidney injury.

Since vascular reactivity to ACh in the thoracic aorta 28 days after reperfusion was markedly attenuated, we examined whether histological changes were present in the thoracic aorta. However, no histological differences in the vascular endothelium or smooth muscle were observed among all groups (Figure 10).

## 4. Discussion

AKI causes remote organ damage due to abnormal interorgan communication between the kidneys and other organs (brain, lungs, heart, liver, intestines, etc.) caused by the temporary or long-term accumulation of uremic toxins, electrolyte imbalance, and excessive fluid retention [15, 16]. In particular, the crosstalk between the kidneys and the heart is known as cardiorenal syndrome and has been the subject of numerous studies. As an example of cardiorenal syndrome, epidemiological studies demonstrated that the incidence of cardiovascular disease, such as heart failure and myocardial infarction, increases after the onset of AKI [17]. It is known that vascular endothelial dysfunction associated with renal injury is not only involved in the development and progression of cardiovascular disease but is also a risk factor for albuminuria and the progression of renal disorder [18]. Therefore, we first evaluated the endothelial function of the thoracic aorta, a large vessel, over time after the onset of AKI.

An important finding in this study was that although the endothelial function of the thoracic aorta was normal at the time when renal function declined due to I/R, endothelial dysfunction progressed thereafter in contrast to the recovery of renal function. However, no histological changes in the thoracic aorta were observed at 28 day after reperfusion. Changes in renal function in the ischemic AKI model are known to be markedly worse immediately after reperfusion, followed by recovery over time [19]. The present ischemic AKI model exhibited a similar tendency in renal function. Both renal arteries and blood vessels distal from the kidney were previously assessed regarding the relationship between AKI and vascular function. Renal arteries after AKI were reported to induce excessive vasoconstriction by adenosine, endothelin, and prostaglandins, in addition to NO trapping by the overproduction of oxidative stress due to promotion of the renin-angiotensin-aldosterone system [20]. Furthermore, the renal arcuate artery of rats subjected to 60 min of bilateral renal ischemia had markedly reduced responsiveness to ACh at 1 hr after reperfusion [21]. Regarding the thoracic aorta, in the ischemic AKI model with 60 min of renal ischemia (45 min of ischemia in this experiment), there was a significant decrease in responsiveness to ACh and a slight decrease in responsiveness to SNP at 5 days after reperfusion [22]. Thus, in the kidney, which is the primary site of AKI, vascular endothelial dysfunction occurs early after reperfusion; on the other hand, in the remote vessels (i.e., thoracic aorta), endothelial dysfunction occurs in the same manner; although, the time of occurrence is later than in the primary site.

ROS, such as O_2_^−^, are highly involved in the development and progression of diseases [23]. In vascular tissues in particular, O_2_^−^ reacts with NO, resulting in the inactivation of NO [24]. The overproduction of O_2_^−^ after renal I/R has been reported to occur not only in renal tissue but also in remote vessels. As an example, excessive O_2_^−^ production occurred in the gracilis artery 5 weeks after the onset of AKI, and O_2_^−^ levels were normalized by administration of the NADPH oxidase inhibitor apocynin [25]. This supports this study, in which O_2_^−^ production increased in vessels distal to the kidneys after the onset of AKI despite the recovery of renal function. Thus, excessive O_2_^−^ production in vascular tissue may play a role in the loss of vascular endothelial function in a remote lesion that occurs after AKI.

The influence of IS on vascular tissue has been reported in many studies. For example, we previously reported that IS causes vascular endothelial dysfunction by trapping NO through O_2_^−^ production induced by the activation of NADPH oxidase [9]. Furthermore, the responsiveness of the thoracic aorta to ACh was significantly attenuated in the 5/6 nephrectomized CKD model with a marked increase in the serum IS concentration. In addition, the administration of AST-120 significantly improved the responsiveness to ACh by suppressing the serum IS concentration [10]. In the ischemic AKI model we used in this study, blood IS levels increased markedly with the decline in renal function 1 day after reperfusion and then slightly decreased along with the recovery of renal function. Therefore, we hypothesized that a transient increase in IS after I/R is involved in the endothelial dysfunction associated with AKI and investigated the effects of AST-120. AST-120 can suppress blood IS levels by adsorbing indole, a precursor of IS, in the intestine [26]. IS is known to function in the progression of renal injury, and administration of AST-120 before ischemia was reported to prevent the development of ischemic AKI [11]; administration before ischemia is not suitable for assessing the impact of IS itself. In addition, in that study, AST-120 administration at 3, 6, and 24 hr after reperfusion resulted in a significant decrease in blood IS concentration without improvement in renal function. Referencing this study, we also administered AST-120 at 3, 6, and 24 hr after reperfusion. In this experiment, similar to the previous study, AST-120 administration markedly reduced the blood IS concentration. However, vascular endothelial function did not improve 28 days after reperfusion even though the increase in blood IS concentration early after reperfusion was suppressed by AST-120. Furthermore, there was no association between blood IS levels 1 day after reperfusion and responsiveness to ACh or O_2_^−^ production 28 days after reperfusion. Taken together, the contribution of the temporarily increased IS to the decline in endothelial function of the thoracic aorta associated with ischemic AKI may not be great.

In this study, AST-120 administration after reperfusion significantly suppressed the increase in plasma IS level associated with I/R treatment, but it was not restored to the sham level. Importantly, even if AST-120 is administered prior to ischemia, the serum IS levels are different from those in the sham group [11, 27]. AST-120 is a spherical adsorbent and therefore cannot eliminate IS that is present in the blood before ischemia or at the onset of reperfusion. Thus, it is difficult to suppress the increase in plasma IS levels associated with AKI unless AST-120 is administered long before ischemia. On the other hand, AST-120 administration after reperfusion was reported to improve cardiac dysfunction associated with AKI without reversing the serum IS level to that in the sham group [28]. Therefore, we hypothesized that vascular endothelial dysfunction can be improved without completely normalizing the plasma IS level. In addition, as mentioned above, administration of AST-120 to CKD model rats markedly improved vascular endothelial dysfunction, but the serum IS level was reportedly only suppressed by approximately 56% and remained higher than that in sham rats [10]. In our studies, the plasma IS level 1 day after reperfusion was suppressed by approximately 62% by AST-120 administration, which is similar to the degree of suppression reported in [10], but vascular endothelial function was not improved. Taken together, the contribution of IS to vascular endothelial dysfunction may be considerably different between the early or temporary increase in IS in ischemic AKI and the chronic increase in CKD.

Excessive inflammatory response has been focused on as one of the causes of distant organ damage after AKI [29]. Abnormal promotion of the inflammatory cascade after the onset of AKI was reported to markedly increase plasma IL-6, leading to lung damage and other problems [30]. Of note, the administration of CNI-1493, which inhibits the release of macrophage-derived inflammatory cytokines, ameliorated the increased vascular permeability of pulmonary arteries that occurs after the onset of AKI without affecting renal function [31]. In addition, as activated neutrophils produce excessive amounts of ROS at the site of inflammation [30], it is possible that the excessive inflammatory response after AKI played a role in the vascular endothelial dysfunction accompanied by increased O_2_^−^ production at the site distal from the kidney. The relationship between inflammatory response and vascular dysfunction after AKI should be investigated in the future.

There are three limitations in this study. The first is that we administered AST-120 only early phase after reperfusion. The second limitation is that we were unable to address whether there is a threshold level for IS to cause vascular endothelial dysfunction. The third limitation is that we have not evaluated the association of IS with vascular endothelial dysfunction in other ischemic AKI models, including the bilateral renal I/R model and unilateral renal I/R model with delayed contralateral nephrectomy. These issues need to be considered in the future.

## 5. Conclusions

In summary, impaired renal function due to AKI recovered over time; however, the endothelial function of the thoracic aorta deteriorated from the time when renal function shifted toward recovery. In addition, administration of the spherical adsorbent AST-120 early after reperfusion was ineffective against the vascular dysfunction associated with ischemic AKI, suggesting the possibility that the temporarily increased IS is not a major contributor. Moreover, it is important to examine whether the impaired vascular endothelial function in large vessels after ischemic AKI plays a role in the development of cardiovascular diseases and the transition to CKD.

## Figures and Tables

**Table 1:** Summary of the least squares regression analysis for significant relationships in vascular dysfunction and O_2_^−^ overproduction.

Variable	Reactivity to ACh		O_2_^−^ production	
	*t* value	*P* value	*t* value	*P* value
(Intercept)	22.38	<0.001	3.738	0.002
IS level	-1.068	0.301	0.581	0.571
I/R (+/-)	-2.959	0.009	3.929	0.002
*R* ^2^	0.578		0.744	
Adjusted *R*^2^	0.528		0.705	

## Data Availability

All data used to support the findings of this study are included within the article.
